# Supplementary figures and images for: Relationship between protein conformational stability and its immunogenicity when administering antigens to mice using adjuvants—Analysis employed the CH2 domain in human antibodies
Source: PLoS One. 2024 Jul 22;19(7):e0307320. doi: 10.1371/journal.pone.0307320 (PMC11262634; doi:10.1371/journal.pone.0307320)

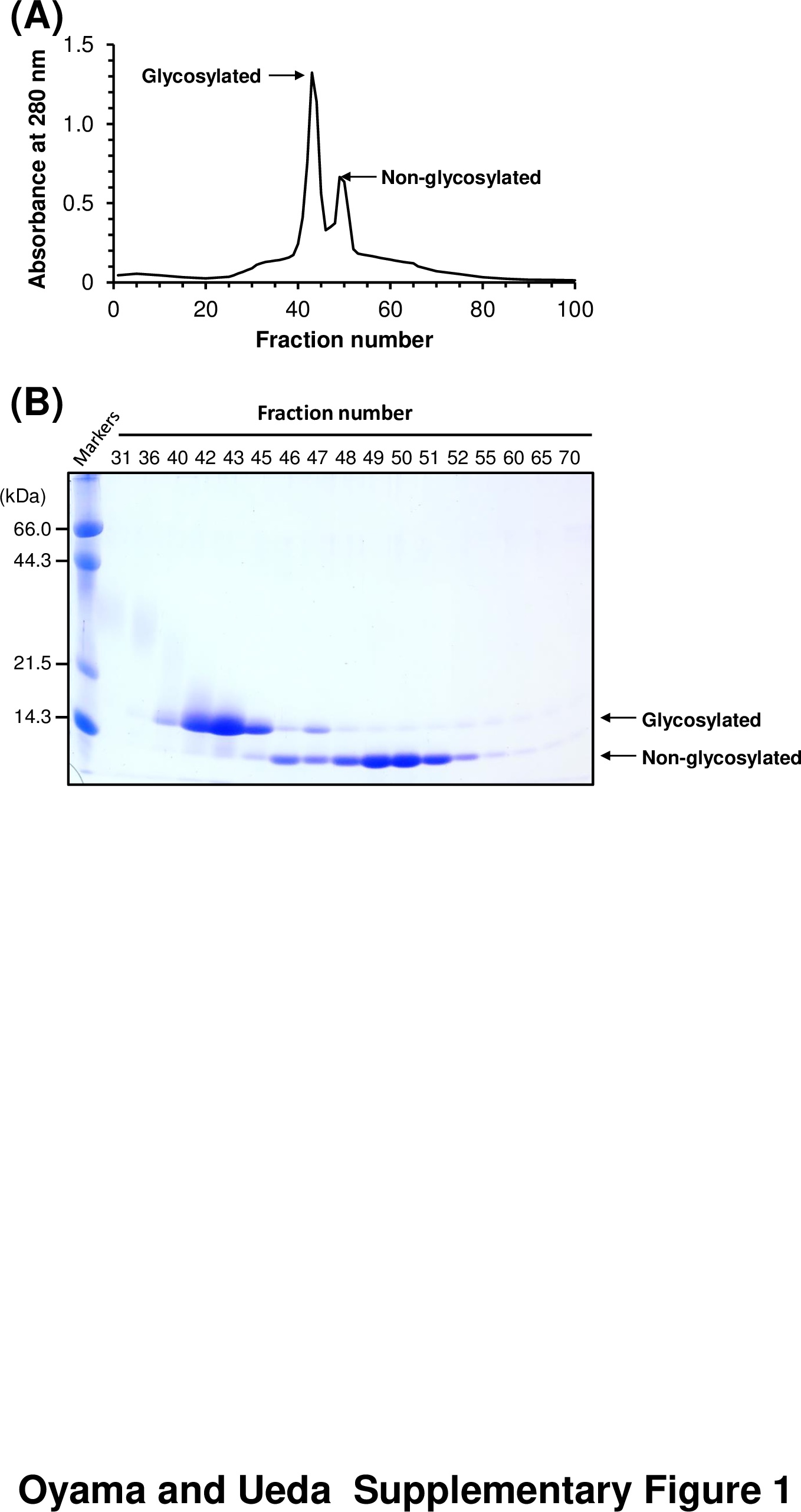

Supplement: S1 Fig — The CH2 domain was expressed in Pichia pastoris and purified following the protocol described by Oyama et al. (BBRC, 2021). In summary, the supernatant underwent purification using a TOYOPEARL SP-650M cation exchange chromatography column (2.6 cm × 2 cm). Moreover, the protein fractions were further purified using a TOYOPEARL SP-650M cation exchange chromatography column (1.6 cm × 100 cm). The eluted fractions were monitored at 280 nm (A) and analyzed utilizing SDS-PAGE (B). The study carried out by Oyama et al. (J. Biochem., 2021) demonstrated that the molecular weight of the expressed protein corresponded to the theoretical weight of the CH2 domain. This discovery confirms that the expressed protein accurately represents the CH2 domain. While these figures show similarities to those illustrated in the research conducted by Oyama et al. (J. Biochem. 2021), these figures differed from those reported in the study conducted by Oyama et al. (J.Biochem., 2021). (JPG) [file pone.0307320.s001.jpg]
