# Supplementary material for: Relationship between protein conformational stability and its immunogenicity when administering antigens to mice using adjuvants—Analysis employed the CH2 domain in human antibodies
Source: PLoS One. 2024 Jul 22;19(7):e0307320. doi: 10.1371/journal.pone.0307320 (PMC11262634; doi:10.1371/journal.pone.0307320)
Supplement: Attachment — Submitted filename: Response to authors-PONE-D-23-43523 .docx [file pone.0307320.s003.docx]

**Response to authors:**

**PONE-D-23-43523**
Relationship between protein conformational stability and its immunogenicity when administering antigens to mice using adjuvants - Analysis employed the CH2 domain in human antibodies -
Dr. Tadashi Ueda

General comment: The authors have made an attempt to elucidate the stability of proteins and their immunogenicity. Overall, the rationale and study design are quite interesting. However, further clarification and improvements in manuscript writing are needed.

Specific comments:

Abstract:

1. The abstract should be based solely on your findings, e.g., anti-drug antibodies are not directly related to this study.
2. The abstract should not include discussion points, such as “The findings, in conjunction with a previous study, indicate that protein stability plays a crucial role in eliciting an immune response in mice through the binding of protein antigens to B cell receptors on APCs.”
3. There is no supporting data from this study for “It is important to note that the optimal stability of antigens for antibody production is anticipated to vary depending on the use of different adjuvants in animal experiments.”

Introduction:

4. The mechanism of action of Alum and IFA/CFA is required, as they might have different immune stimulation pathways, which could lead to differences in immune induction capability (not solely due to the effect of protein stability).
5. RNase A and RNase S were not mentioned.
6. Information about mut20 (mutation position, changes in disulfide bonds, etc.) and its conformation (compared to the wild-type) must be included.

Materials and Methods:

7. Producing mut20 in yeast could potentially yield a glycosylated protein. How did the authors avoid the effect of the glycosylation status of these two proteins on antibody induction capability?
8. The statement “All other chemicals used in the study were of the highest commercial quality available” is vague. How can the authors ensure they were the best quality on the market?
9. The immunization schedule was quite short (1-week intervals). For protein (and other platform) vaccine immunization, at least a 2-week interval is usually employed to allow immune cell maturation.
10. The approved animal protocol number should be included.
11. Why was the intraperitoneal route used instead of the intramuscular route? This does not reflect the standard immunization procedure.
12. Why were different protein amounts used for adjuvanted and non-adjuvanted groups (30 vs. 100 micrograms)? We cannot directly compare the magnitude of the antibody response as the vaccine doses differ.
13. What is the reason for using CFA in the first immunization and IFA for the booster? Please explain and state this in the manuscript.
14. Salt concentrations in buffers must be indicated as their final concentrations (mol/liter, molar), not in grams.
15. Line 105, what is the initial dilution, and how did you dilute the tested serum?
16. ELISA: The methods for stopping the reaction and reporting readouts are missing. Was the background subtracted?

Results:

17. For all results sections, please do not repeat the introduction and method. Authors must directly explain the obtained results concisely (important). Also, the discussion should be stated separately in the discussion section.
18. For better understanding, CH2, mut20, RNase A, and RNase S should be indicated in Figures 3C-3F.
19. Again, authors never mentioned and explained about RNase A/RNase S; it suddenly appeared in lines 165-177.
20. Where is the data to support the statement? “When examining the MHC-peptide complex in APCs and T cell presentation, it is suggested that the immunogenicity of RNase A compared to RNase S is not solely determined by the degree of protease digestion of the RNases.”

Discussion:

21. More discussion is needed on topics other than protein stability, including adjuvant properties. Moreover, as the authors tested in BALB/c mice, which is an inbred strain with a restricted TCR and MHC repertoire, further investigation in an outbred mouse strain may be needed to confirm your findings.
22. What is the explanation for the opposite IgG results when the adjuvant is changed (Figure 1B)?
