# Supplementary material for: Relationship between protein conformational stability and its immunogenicity when administering antigens to mice using adjuvants—Analysis employed the CH2 domain in human antibodies
Source: PLoS One. 2024 Jul 22;19(7):e0307320. doi: 10.1371/journal.pone.0307320 (PMC11262634; doi:10.1371/journal.pone.0307320)
Supplement: Attachment — Submitted filename: Response to authors-PONE-D-23-43523_F.docx [file pone.0307320.s004.docx]

Specific comments:

(Abstract)

**Comment 1**

The abstract should be based solely on your findings, e.g., anti-drug antibodies are not directly related to this study.

**Response:** Based on your comment, we have revised the abstract to focus solely on the findings directly related to this study. We have removed the sentences about the anti-drug antibodies.

**Comment 2**

The abstract should not include discussion points, such as “The findings, in conjunction with a previous study, indicate that protein stability plays a crucial role in eliciting an immune response in mice through the binding of protein antigens to B cell receptors on APCs.”

**Comment 3**

There is no supporting data from this study for “It is important to note that the optimal stability of antigens for antibody production is anticipated to vary depending on the use of different adjuvants in animal experiments.”

**Response:** Thank you for your comments. We have revised the abstract accordingly to remove discussion points. Instead, the findings of this study have been incorporated into the Abstract section of the revised manuscript.

(Introduction)

**Comment 4**

The mechanism of action of Alum and IFA/CFA is required, as they might have different immune stimulation pathways, which could lead to differences in immune induction capability (not solely due to the effect of protein stability).

**Response:** Thank you for your valuable feedback. We comprehended the supplementary explanation regarding the mechanism of action of Alum and IFA/CFA, because the outcome of this study was found to be associated not only with the impact on protein stability but also with a biological factor, specifically the mechanism of action, of CFA/IFA. In the revised manuscript, we have incorporated a description on the mechanism of action of Alum and CFA/IFA, along with an analysis of the impact of CFA/IFA on protein stability, into the text (page 3 - page 4 line 62 - line 76 in the Introduction section). Based on the literature (Biliau and Matthys, J. Leukoc. Biol., 2001; Lim, Int. Immunopharmacol., 2003) referenced in the revised manuscript, it has been documented that the presence of inactivated *Mycobacterium tuberculosis* in CFA influences immunological responses. This includes the stimulation of T-lymphocytes to adopt a Th1 profile, leading to a robust delayed type hypersensitivity reaction against autoantigens and an upregulation of Toll-like receptor 2 expression. Hence, to eliminate the impact of inactivated *Mycobacterium tuberculosis* on IgG expression following the administration of an antigen with an adjuvant, we conducted a supplementary series of animal experiments utilizing only IFA as the adjuvant. Consequently, our findings indicate that the production of IgG against mut20 was higher compared to that of CH_2_ domain when administered with IFA as an adjuvant, suggesting that a pattern that was consistent when administering them with CFA/IFA. Therefore, our results suggest that the variations in IgG production observed during antigen administration with Alum versus CFA/IFA were not influenced by the biological activity of inactivated *Mycobacterium tuberculosis*. Instead, they were attributed to the impact of the adjuvant on protein stability. Therefore, the revised manuscript includes the outcomes of a sequence of animal experiments that employed IFA solely as an adjuvant (see Figure 1B and page 9, line 163 - line 169) in the revised manuscript. Additionally, the aforementioned description, in conjunction with the references, has been incorporated into the Discussion section of the revised manuscript (page 11, lines 212-215) .

**Comment 5**

RNase A and RNase S were not mentioned.

**Response:** We acknowledge the feedback provided regarding the description of RNase A and RNase S used in our study. Delamarre et al. (Science, 2005; J. Exp. Biol. Med. 2006) showed that the administration of RNase A with Alum in mice led to increased IgG production in comparison to the administration of RNase S. Additionally, it was observed that RNase A exhibited greater stability than RNase S. These results seem to conflict with the outcomes of our investigation on the CH_2_ domain in animal experiments involving Alum. In response to your inquiry, information concerning RNase A and RNase S has been integrated into the introduction (page 5, line 89 - line 100). By incorporating these proteins in our research, we can explore the importance of conformational stability on the immune response when administering protein antigens to mice with Alum adjuvant.

**Comment 6**

Information about mut20 (mutation position, changes in disulfide bonds, etc.) and its conformation (compared to the wild-type) must be included.

**Response:** Thank you for acknowledging the importance of providing thorough information on mut20 and its conformational changes in relation to the wild-type protein. In our response, we have furnished comprehensive details concerning mut20, including its mutation position and alterations in disulfide bonds, in the pertinent section of the manuscript (page 4, line 81 - line 83) in the revised manuscript.

(Materials and Methods)

**Comment 7**

Producing mut20 in yeast could potentially yield a glycosylated protein. How did the authors avoid the effect of the glycosylation status of these two proteins on antibody induction capability?

**Response:** We appreciate the constructive feedback received on our manuscript. In numerous instances, glycosylated and non-glycosylated proteins are acquired through the yeast expression system. In our prior investigation (Oyama et al., J Biochem, 2021; Biophys. Biochem. Res. Commun, 2021), we effectively isolated the glycosylated and non-glycosylated CH_2_ domain through cation exchange chromatography (described in below figures). In this study, we utilized the non-glycosylated protein to mitigate any potential impact of protein glycosylation status on antibody induction capacity.

Cation exchange chromatography of CH_2_ domain.

**Comment 8**

The statement “All other chemicals used in the study were of the highest commercial quality available” is vague. How can the authors ensure they were the best quality on the market?

**Response:** Based on your feedback, we altered the sentence of “All other chemicals used in the study were of the highest commercial quality available” to that of “All the other reagents and chemicals used in the study were of analytical grade or higher quality” (page 6, line 107 - line 108) in the revised manuscript.

**Comment 9**

The immunization schedule was quite short (1-week intervals). For protein (and other platform) vaccine immunization, at least a 2-week interval is usually employed to allow immune cell maturation.

**Response:** Thank you for your valuable feedback. We recognize that our immunization schedule, with 1-week intervals, may appear shorter when compared to the conventional practice, which typically involves a 2-week interval for protein vaccine immunization. Our immunization protocol was developed based on methodologies previously established in our laboratory (Ohkuri et al., J. Immunol, 2010; Nakamura et al., Biochim. Biophys. Acta, 2016, Ohkuri et al., Biochem. Biophys. Rep., 2019, and Masuda et al., Front. Immunol., 2022). Despite the unconventional schedule, this protocol has consistently produced monoclonal IgGs with strong binding abilities, resulting in a sub-nanomolar K*_D_* of the Fab-antigen complex during the hybridoma generation process. Despite concerns about immune cell maturation, the generation of high-affinity antibodies suggests that immune cells have undergone effective maturation in accordance with our immunization protocol. This observation indicates that the effectiveness of our methodology, specifically the relatively short interval of immunization (1-week intervals), has little effect on inducing strong immune responses.

**Comment 10**

The approved animal protocol number should be included.

**Response:** Based on your comment, we have included the number, A21-053-0 (line 115 on page 6) in the revised manuscript.

**Comment 11**

Why was the intraperitoneal route used instead of the intramuscular route? This does not reflect the standard immunization procedure.

**Response:** Thank you for your valuable feedback. The intraperitoneal route was selected based on several considerations. Firstly, it has been widely utilized in previous research studies that investigate similar research questions, thereby enhancing the ability to compare and maintain consistency across experiments. Furthermore, Freund's adjuvant, which was employed in our study, is recommended for intraperitoneal administration in the manufacturer's guidelines. Moreover, Delamarre et al. (2006) illustrated in their research published in the Journal of Experimental Medicine, which paper is closely related in this study, that intraperitoneal administration resulted in increased antibody production in comparison to intramuscular administration and was also observed that the method of administration did not have a significant impact on protein stability and immunogenicity. We think that these suggests that the intraperitoneal route can be considered a suitable option without compromising the integrity of our experimental results.

**Comment 12**

Why were different protein amounts used for adjuvanted and non-adjuvanted groups (30 vs. 100 micrograms)? We cannot directly compare the magnitude of the antibody response as the vaccine doses differ.

**Response:** We appreciate the constructive feedback received regarding our manuscript. The main aim of our study was to evaluate the influence of protein stability on immunogenicity while maintaining consistent adjuvant conditions. To accomplish this, varying protein dosages were utilized for the adjuvanted and non-adjuvanted groups to guarantee equivalent levels of antibody production. This approach was employed to consider the unique immune stimulation capacities of various adjuvants and to enable valid comparisons between different groups. While acknowledging that this approach may restrict direct comparisons of antibody responses among different adjuvant groups because of variations in protein dosage, it enables us to evaluate the relative immunogenicity of proteins with different stability levels under standardized adjuvant conditions.

**Comment 13**

What is the reason for using CFA in the first immunization and IFA for the booster? Please explain and state this in the manuscript.

**Response:** Thank you for your comment. This sequential administration adheres to a standard protocol in which CFA, recognized for its ability to induce strong inflammation, is used initially, followed by IFA for enhancing immune responses.

**Comment 14**

Salt concentrations in buffers must be indicated as their final concentrations (mol/liter, molar), not in grams.

**Response:** Based on the feedback provided, we converted grams to molar or percentage units, highlighting characters in questions in red color.

**Comment 15**

Line 105, what is the initial dilution, and how did you dilute the tested serum?

**Response:** We apologize for the lack of clarity concerning the dilution factor utilized in ELISA. In our investigation, serum samples from animals underwent varying dilution factors in ELISA to accommodate potential discrepancies in antibody production across distinct adjuvant groups. The methodology section has been revised as indicated (page 7 line 130 - line 131) in the revised manuscript.

**Comment 16**

ELISA: The methods for stopping the reaction and reporting readouts are missing. Was the background subtracted?

**Response:** In our ELISA protocol, we employed ABTS (2,2'-azino-bis(3-ethylbenzothiazoline-6-sulfonic acid)) as the substrate for the horseradish peroxidase (HRP). In contrast to other substrates like TMB (3,3',5,5'-tetramethylbenzidine), ABTS is characterized by a slower reaction rate and is commonly employed without the requirement of a stop solution. Consequently, the reaction was permitted to continue until the intended endpoint was achieved. To guarantee precision in our measurements, the background absorbance was subtracted from the raw data before conducting the analysis.

(Results)

**Comment 17**

For all results sections, please do not repeat the introduction and method. Authors must directly explain the obtained results concisely (important). Also, the discussion should be stated separately in the discussion section.

**Response:** We are grateful for the constructive feedback provided on our manuscript. Based on your feedback, we have removed redundant sections and focused solely on presenting the obtained results concisely.

**Comment 18**

For better understanding, CH2, mut20, RNase A, and RNase S should be indicated in Figures 3C-3F.

**Response :** We acknowledge the recommendation to provide labels for CH_2_ domain, mut20, RNase A and RNase S in Figures 3C-3F to enhance clarity. 　The figures have been updated accordingly.

**Comment 19**

Again, authors never mentioned and explained about RNase A/RNase S; it suddenly appeared in lines 165-177.

**Response:** Thank you for the valuable feedback that was provided.　As described in response to comment 5, we employed RNase A and RNase S.　 These proteins are known to produce results that differ from those of the CH_2_ domain in animal studies involving Alum. The aim of this study is to investigate RNase A and RNase S, as mentioned in the Introduction and result section (page 10, line 190- line 191) in the revised manuscript.

.

**Comment 20**

Where is the data to support the statement? “When examining the MHC-peptide complex in APCs and T cell presentation, it is suggested that the immunogenicity of RNase A compared to RNase S is not solely determined by the degree of protease digestion of the RNases.”

**Response:** We appreciate the constructive feedback received on our manuscript. We have included the comprehensive description (page 10, line 196 - line 202) in the revised manuscript.

.

(Discussion)

**Comment 21**

More discussion is needed on topics other than protein stability, including adjuvant properties. Moreover, as the authors tested in BALB/c mice, which is an inbred strain with a restricted TCR and MHC repertoire, further investigation in an outbred mouse strain may be needed to confirm your findings.

**Response:** We appreciate the constructive feedback received on our manuscript. We have incorporated the mechanism of action of Alum and CFA/IFA adjuvant as described in Introduction (page 3 - page 4, line 61 - line 75) in the revised manuscript. Moreover, in this study, we included the results of animal studies utilizing only IFA as an adjuvant to further clarify the impact of inactivated *Mycobacterium tuberculosis* on the immune response (Fig. 1B). We have comprehended the significance of your comment suggesting that additional research in an outbred mouse strain may be necessary to validate our findings, because we use mut20, which contains two amino acid residues that differ from the CH_2_ domain. Regrettably, our academic department does not possess the essential resources required to conduct animal experiments utilizing outbred mouse strains. Under the specified circumstances, it was observed that the deamidation of asparagine within the T cell epitope of hen egg lysozyme significantly influenced T cell recognition (McAdam et al. J. Exp. Med. 2001). Moreover, Tsujihata et al. (Mol. Immunol. 2001) identified a crucial single point mutation in the T cell epitope of hen egg lysozyme that is necessary for T cell recognition. This mutation resulted in a restricted generation of IgG antibodies targeting the antigen. Based on these reports, when the two point mutations between CH_2_ and mut20 are located within the T cell epitope of the CH_2_ domain, it is hypothesized that there will be a minimal IgG response subsequent to the administration of antigens to mice utilizing Alum or CFA/IFA. However, the production of IgG was observed to investigate the relationship between protein conformational stability and immunogenicity without the need to use an outbred mouse strain.

In the original manuscript, our study focused on examining the influence of the intrinsic global structure that can efficiently bind to B-cell receptors (BCR) on APCs. In a prior study conducted by Masuda et al. Front Immunol. 2022, the administration of mice with the SARS-CoV-2 S-protein using CFA/IFA adjuvant did not result in the production of neutralizing antibodies. The receptor binding domain (RBD) within the trimeric S-protein undergoes hinge-like conformational movements that transiently hide or expose the receptor binding determinants and the predominant state of the trimer, which facilitates evasion to cells, involves one of the three RBDs being rotated upwards in the receptor-accessible conformation, as demonstrated using Cryo-EM (Wrapp et al. Science 2020). Furthermore, Cryo-EM analysis revealed that a neutralizing antibody was bound to one of the three RBD and caused it to rotate upwards (Barnes et al., Cell 2020). Hence, it is plausible that the destabilizing impact caused by mineral oil from CFA or IFA could influence the hinge-like conformational changes of the RBD in the trimeric S-protein. The Discussion section of the revised manuscript explores the potential impact of CFA/IFA adjuvants on the hinge-like conformational movements of the RBD in the trimeric S-protein from SARS-CoV-2 (page 11- page 12 line 221 - line 229) in the revised manuscript.

**Comment 22**

What is the explanation for the opposite IgG results when the adjuvant is changed (Figure 1B)?

**Response:** We appreciate the constructive feedback received on our manuscript. In the process of antibody production, the successful binding of the antigen to the BCR and the subsequent presentation of antigen peptides to the TCR as a peptide-MHC are essential steps. When utilizing PBS and Alum, the intact CH_2_ domain and mut20 exhibit conformational stability, enabling effective binding with BCR. The quantity of digestion peptides derived from the CH_2_ domain exceeded that of mut20. Consequently, the immune response induced by the CH_2_ domain was greater compared to that of the mut 20 when administered with Alum and PBS. Freund's adjuvant has the ability to reduce the conformational stability of antigens due to its hydrophobic characteristics. Therefore, an intact CH_2_ domain may not possess adequate stability to effectively bind with the BCR in the presence of Freund's adjuvant. However, mut20 maintains effective binding to the BCR even when Freund's adjuvant is present, possibly aided by enhanced peptide fragment generation resulting from the protein-destabilizing impact of Freund's adjuvant. Hence, the immune response triggered by the CH_2_ domain was found to be lower when Freund's adjuvant was utilized compared to mut20. In an effort to improve the understanding of our findings, we have provided a description (page 11, lines 212-215) in the Discussion section and have made adjustments to Figure 4 to enhance its clarity in the revised manuscript.
