# Supplementary material for: Relationship between protein conformational stability and its immunogenicity when administering antigens to mice using adjuvants—Analysis employed the CH2 domain in human antibodies
Source: PLoS One. 2024 Jul 22;19(7):e0307320. doi: 10.1371/journal.pone.0307320 (PMC11262634; doi:10.1371/journal.pone.0307320)
Supplement: Attachment — Submitted filename: Response to Reviewers .docx [file pone.0307320.s005.docx]

**Comment 7**

Please include the results pertaining to the isolation of non-glycosylated proteins. It would be appropriate to add these details in the supplementary data section. Additionally, clarify how you confirmed the identity of the expressed protein as CH2. Were Western blotting techniques used for this purpose? Please elaborate.

**Response:** Thank you for your constructive comments. We have included the purification of CH_2_ domain in Supplementary Figure 1 and explained why we used non-glycosylated form on page 9, line 176-182. Additionally, we confirmed the identity of the expressed proteins as the CH_2_ domain using MALDI-TOF-MS measurements, as detailed in our previous study (Oyama et al., JB. 2021) on page 9, line 176-182.

Comments 9 and 11

It is necessary to add the reference for the animal protocol that was previously referred to by the authors. This will help in maintaining the integrity of the experimental framework.

Response: Thank you for your valuable feedback. According to the provided comment, initially, the initial rationale for selecting intraperitoneal injection as the method of administration in the animal protocol of our previous studies by Ohkuri et al., J. Immunol, 2010 [17], Nakamura et al., BBA, 2016 [18], Masuda et al., Front. Immunol., 2022 [21], Oyama et al., BBRC, 2021 [36], and Ohkuri et al., BBR, 2019 [38] followed the route of administration utilized in the pioneering study by Delamarre et al., J. Med. Exp.2006 [19] in the revised manuscript text (page 6, lines 118-122). Moreover, we have explicated the interval immunization protocol currently employed, aligning with the methodology established in prior studies by Ohkuri et al., J. Immunol, 2010 [17], Nakamura et al., BBA, 2016 [18], Masuda et al., Front. Immunol., 2022 [21], Oyama et al., BBRC, 2021 [36], and Ohkuri et al., BBR, 2019 [38] (page 7, line 130-133).

**Comments 12 and 13**

I recommend that you integrate a more detailed explanation regarding these specific concerns within the discussion section of the manuscript. Please ensure that relevant references are cited to support your explanations.

**Response:** Based on your comments, we have added a detailed explanation in the discussion section addressing why different protein amounts were used for the adjuvanted and non-adjuvanted groups (to comment 12). It is widely accepted that increasing the antigen dose enhances antibody production, a conclusion supported by our previous studies (Masuda et al., Front. Immunol., 2022 [16]. Therefore, we considered that increasing the antigen dose in the non-adjuvant group is a preferable method for assessing the impact of protein stability on immunogenicity while keeping adjuvant conditions consistent. Additionally, we have added the rationale for using CFA in the first immunization and IFA for the booster in the method section (to comment 13). As previously stated, the content has been integrated as specified, accompanied by proper citation of relevant literature in the Discussion section. (page 12, line 228-241) as well as in the Materials and Methods section（page 7, 126-129) .

**Comment 16**

This detail should be included in the methods section of your paper to provide clarity on the procedures used in your study.

**Response:** Based on the feedback provided, a concise elucidation regarding the utilization of ABTS in the ELISA protocol has been incorporated into the methods section to enhance the comprehensibility of the procedures employed in our research (page 7, line 147 - page 8, line 152).

**Comment 21**

The discussion concerning the choice of mouse strain should remain within the discussion section of the manuscript. This will help readers understand the rationale behind the selection and its relevance to the study outcomes.

**Response:** Thank you for your suggestion. Following your comment, we have incorporated a discussion regarding the choice of mouse strain into the discussion section (page12, line 242- page 13, line 253)
